# Exacerbating exclusion? How the logic of refugee education perpetuates the exclusion of refugees with disabilities in Lebanon

**DOI:** 10.1007/s11159-025-10132-x

**Published:** 2025-03-22

**Authors:** Giada Costantini, Yomna El-Serafy

**Affiliations:** 1 University of Birmingham, Birmingham, UK; 2 University of Cambridge, Cambridge, UK

**Keywords:** Disability, Inclusive education, Humanitarianism, Refugee education, Education in emergencies, Lebanon

## Abstract

This article assesses how the logic of refugee education affects the inclusion of refugees with disabilities. It draws on academic literature, sociological and ethnographic research in Lebanon with refugees with disabilities and refugee education practitioners, and conversations between the authors on practices they witnessed out in the field. They highlight four tensions between how refugee education is conceptualised on the one hand, and the prerequisite logics for disability-inclusive education on the other. First, they historicise the emergence of refugee education, highlighting how the logic of securitisation facilitates the exclusion of refugees with disabilities who fall outside constructs of the “threatening migrant”. Second, they highlight the neoliberal logics shaping funding structures and educational assumptions within refugee education. Ideals of cost–benefit analyses and future employability interact with ableist assumptions to construe refugees with disabilities as less valuable to include. Third, the reliance on vulnerability frameworks leads to disempowering perceptions of disability that conflict with more equitable narratives of diversity and inclusion. Fourth, conflicting temporal pressures are at play between ideas of “emergency” education, which have a temporary and present-oriented focus, and disability-inclusive education, which is developmental and future-oriented. The tensions between the dominant lexicon of refugee education and the philosophy underlying inclusive education contribute to marginalisation, disempowerment and exclusion. This article calls for the refugee education community of scholars and practitioners to engage in critical reflection on how the frameworks within which we work might better support the recognition, inclusion and dignified treatment of refugees with disabilities.

## Résumé

Une exacerbation de l’exclusion ? Comment la logique de l’éducation des réfugiés perpétue l’exclusion des réfugiés en situation de handicap au Liban – Le présent article évalue dans quelle mesure la logique de l’éducation des réfugiés influe sur l’inclusion de ceux qui parmi eux se trouvent en situation de handicap. Il s’appuie sur la littérature scientifique, des recherches sociologiques et ethnographiques menées au Liban auprès de réfugiés en situation de handicap et de praticiens de l’éducation des réfugiés, ainsi que sur des échanges entre les autrices concernant les pratiques qu’elles ont observées sur le terrain. Les autrices mettent en évidence quatre points de tension entre, d’une part, la façon dont l’éducation des réfugiés est conceptualisée et, d’autre part, les logiques préalablement nécessaires à une éducation inclusive pour les personnes en situation de handicap. Premièrement, elles dressent un historique de l’émergence de l’éducation des réfugiés, soulignant comment la logique de sécurisation facilite l’exclusion de ceux d’entre eux en situation de handicap qui échappent à la construction du « migrant menaçant ». Deuxièmement, elles mettent en lumière les logiques néolibérales qui façonnent les structures de financement et les hypothèses éducatives dans l’éducation des réfugiés. Les idéaux des analyses coûts-bénéfices et d’employabilité future interagissent avec des postulats capacitistes, conduisant à considérer les réfugiés en situation de handicap comme moins dignes d’être inclus. Troisièmement, le recours aux modèles de vulnérabilité conduit à des perceptions déresponsabilisantes du handicap, en contradiction avec des récits plus équitables de diversité et d’inclusion. Quatrièmement, des pressions temporelles conflictuelles entrent en jeu entre les idées d’éducation « en situation d’urgence », axées sur le temporaire et le présent, et l’éducation inclusive, évolutive et tournée vers l’avenir, des personnes en situation de handicap. Les tensions entre la terminologie dominante de l’éducation des réfugiés et la philosophie sous-jacente à l’éducation inclusive contribuent à la marginalisation, à la dépossession du pouvoir d’agir et à l’exclusion. L’article appelle la communauté des chercheurs et praticiens du secteur de l’éducation des réfugiés à une réflexion critique sur la manière dont les cadres dans lesquels ils travaillent pourraient mieux soutenir la reconnaissance, l’inclusion et un traitement digne des réfugiés en situation de handicap.

## خلاصة

تفاقم الإقصاء؟ كيف يعيد منطق تعليم اللاجئين إنتاج استبعاد اللاجئين ذوي الإعاقة في لبنان - يقيّم هذا المقال كيف يؤثر منطق تعليم اللاجئين على إدماج اللاجئين ذوي الإعاقة. وينهل من الأدبيات الأكاديمية، والأبحاث السوسيولوجية والإثنوغرافية التي أُجريت في لبنان مع لاجئين من ذوي الإعاقة ومع ممارسي تعليم اللاجئين، كما يستند إلى الحوارات التي أجراها المؤلفون حول الممارسات التي شهدوها ميدانيًا. ويبرز المقال أربع توترات جوهرية بين التصورات السائدة حول تعليم اللاجئين من جهة، والمنطلقات المعرفية الضرورية للتعليم الشامل للإعاقة من جهة أخرى. أولًا، يؤرخ المقال لنشأة تعليم اللاجئين، مسلطًا الضوء على كيفية تسهيل منطق الأمننة لاستبعاد اللاجئين ذوي الإعاقة الذين يقعون خارج تصنيفات "المهاجر المُهدِّد". ثانيًا، يستكشف المقال المنطق النيوليبرالي الذي يشكّل هياكل التمويل والافتراضات التربوية ضمن تعليم اللاجئين، موضحًا كيف تتفاعل تحليلات الكلفة والعائدات مع الافتراضات القادرة بحيث يُنظر إلى اللاجئين ذوي الإعاقة على أنهم أقل قيمة من حيث الإدماج. ثالثًا، يؤدي الاعتماد على أطر الهشاشة إلى تكريس تصورات مُحبِطة للإعاقة تتعارض مع السرديات الأكثر إنصافًا حول التنوع والإدماج. رابعًا، توجد توترات زمنية بين منطق التعليم في حالات "الطوارئ"، الذي يركز على الحاضر ويُفهم على أنه مؤقت، وبين التعليم الشامل للإعاقة، الذي يتسم بطابع تنموي ومستقبلي. تساهم هذه التوترات بين الخطاب السائد في تعليم اللاجئين والفلسفة الكامنة وراء التعليم الشامل في تعميق التهميش، وإعادة إنتاج ديناميات الإقصاء، وإضعاف الوكالة الفردية والجماعية للاجئين ذوي الإعاقة. ويختتم المقال بالدعوة إلى انخراط مجتمع الباحثين والممارسين في تعليم اللاجئين في مراجعة نقدية لإطارات العمل التي نعتمدها، بهدف تعزيز الاعتراف بحقوق اللاجئين ذوي الإعاقة، وضمان إدماجهم العادل، وتمكينهم من معاملة كريمة تتناسب مع كرامتهم الإنسانية.

## Introduction

In times of crisis and forced displacement, education is critical. It provides students with learning continuity, communities of belonging, and improved socioemotional well-being (Dryden-Peterson 2017; Salem 2021). Despite ongoing scholarly discourses on access, quality, and the purposes of refugee education, many refugees continue to be out of school. This is particularly true for refugees with disabilities. Recognising this, the field of refugee education is witnessing a positive trend among international actors advocating for disability inclusion, evident through our research and through the increase in policies and funding targeting refugees with disabilities (INEE 2023; UNICEF 2023; ECW 2022). However, our research suggests that these projects often over-emphasise “access” to schooling at the expense of participation and quality, fall short of the dignifying narratives that support disability inclusion, and consistently fail to achieve their goals (see Trani et al. 2012; Singal 2019). As a result, learners with disabilities continue to be excluded.

Research on the marginalisation of refugees with disabilities highlights physical challenges such as inadequate transportation, dangerous paths to and from school, and poor infrastructure (Karanja 2009; Smith-Khan 2013; Smith-Khan and Crock 2018). While these physical barriers are important, we argue that the contemporary lexicon of refugee education contains integral assumptions which lead to the exclusion of refugees with disabilities, even as international actors seek to include them. In this article, we reflect on four such problematic ideas within the contemporary discourse on refugee education.

Historically, the growth of the fields of refugee education and disability studies has been separated by disciplinary boundaries in both theory and practice (Walton et al. 2020). As these fields are now beginning to come together in academic scholarship, it is an opportune moment to reflect on how assumptions within refugee education may exacerbate exclusion for people with disabilities. This article advocates for a critical shift in refugee education, highlighting the need to engage with disability studies (Gill and Schlund-Vials 2014) to acknowledge and address the ableist and exclusionary assumptions often obscured by its “benevolent façade” (Donini 2010, p. S221). We critically examine how the dominant logic of refugee education affects the inclusion of refugees with disabilities by drawing on sociological and ethnographic research conducted in Lebanon, alongside academic literature and field-based observations. Highlighting the tensions between prevailing refugee education frameworks and the foundational principles necessary for disability-inclusive education, this article explores historical, neoliberal, vulnerability-based and temporal barriers to inclusion. Ultimately, it calls for a re-evaluation of the conceptual frameworks shaping refugee education to promote inclusive education for all refugees.

This article is based on qualitative doctoral research conducted separately by the authors with refugees^1^ with disabilities in Lebanon. The first author’s work (Costantini 2025) draws on empirical data collected over two months of fieldwork in Lebanon in 2022, consisting of semi-structured interviews with Syrian refugee children and young people with various different types and severities of disabilities and their families. The research also includes semi-structured interviews conducted remotely with representatives of organisations for people with disabilities (OPDs), international and Lebanese non-governmental organisations (NGOs), civil society organisations and United Nations (UN) agencies over a period of four months during the same year. The study employs *critical disability theory* as both a conceptual framework and a research methodology, and *constructivist grounded theory* as a data analysis tool.

The second author’s research (El-Serafy, forthcoming-a) consists of 10 months of ethnographic research with blind refugees in Lebanon, their families and teachers, OPDs, formal and non-formal schools and international and non-governmental organisations between 2020 and 2023. The study is based on a phenomenological epistemological approach employing the methodology of portraiture (Lawrence-Lightfoot and Hoffman Davis 1997) to develop in-depth portraits of the ways in which blind refugees experience education in Lebanon.

Our respective research highlights how refugees with disabilities and their caregivers in Lebanon are often challenging the exclusionary logics of refugee education and proposing alternatives which might inform a more inclusive approach (Sauer 2012).

The present work results from a synthesis of our individual research efforts, professional experience working in humanitarian contexts, relationships built with our participants, a review of the literature, and ongoing discussions related to the research topic. Our article is conceptual in nature. While we recognise that refugee education logics manifest differently across contexts, with significant variation in practice, refugee education is largely conceptualised and operationalised within a Western-dominant framework. This framework, heavily influenced by major international actors, has had a profound impact on the global refugee education landscape – a dynamic we specifically critique.

## Refugee education in Lebanon

Since the onset of war in 2011, Syria has come to represent the world’s largest instance of forced displacement (UNHCR 2022). Lebanon is the second-largest host state of Syrian refugees and the largest host state of refugees per capita, hosting approximately one refugee for every five citizens (ibid.). Lebanon is currently estimated to host more than 1.5 million refugees from Syria, half of whom are of school age (Hamadeh 2019). To meet Syrian refugees’ educational needs, the Lebanese Ministry of Education and Higher Education (MEHE), in collaboration with UN agencies, has implemented the “Reaching All Children with Education” programme (RACE I and RACE II). Some students also attend non-formal schools run by NGOs, which are typically not recognised by the Lebanese government (Crul et al. 2019; Shuayb et al. 2014). Many refugees continue to be out of school, and when they do enrol, they are likely to have low learning outcomes, high dropout rates (Jalbout 2015) and poor psychosocial conditions (Shuayb and Ahmad 2021).

Since educational experiences are so varied, there is no monolithic “logic” at play. Instead, there are a multitude of actors who conceptualise refugee education differently. We use the term “refugee education” in its broadest sense to encompass the variety of educational experiences and pathways of Syrian refugees, particularly focusing on the organisational and governmental provision which targets them. We acknowledge that this does not constitute a comprehensive reflection on refugee education, and that there are attempts to conceptualise refugee education through a social justice lens (Dryden-Peterson et al. 2019). Our aim, here, is to examine key tenets of the dominant Western model of refugee education which is prominent in our research.

Of the Syrian refugees living in Lebanon, approximately 22.8% are estimated to have a disability (UNHCR et al. 2017). Studies suggest that these refugees face increased exclusion from social services, including education (CRC 2017). Some data suggest that refugees with disabilities in Lebanon are more likely to be excluded from schools than either refugees without disabilities or non-refugees with disabilities (Combaz 2018; HRW 2018; UNHCR et al. 2017).

## Disability and inclusive education

We rely on the biopsychosocial model to understand disability (WHO 2002), moving beyond the antiquated medicalised construction of disability as an individual defect. We understand disability as a continuum and a relational reality, which “is ontologically, spatially, temporally, materially, discursively, culturally, socially, politically and economically contingent” (Saltes 2013, p. 56). This framework acknowledges the biological reality of disability, while also recognising society’s complicity in exclusionary practices, as well as the psychological dimension and experiential background of people with disabilities. Audre Lorde (1984 [1982]) writes that “we do not live single-issue lives” (cited by Saia et al. 2023, p. 4), and David Hosking points out that “everyone is multidimensional” (Hosking 2008, p. 10). Disability is not located in an individual’s physical impairment alone, but is the outcome of the interaction between their impairment and various contextual factors, both environmental and personal.

Our research involves people with different types and severities of disabilities and different environmental conditions. This was a frequent topic of reflection between us in developing this article. A crucial cross-cutting problem in the field of refugee education is its homogenisation of disability in planning and reporting. This oversimplification leads to the construction of a binary model of disability: either one has a disability or one does not. This leads to the inclusion of some people with disabilities and the exclusion of others. As a result, those who are excluded are further marginalised and rendered invisible.

In line with this understanding of disability, inclusive education refers to equitable and quality education for every child, regardless of the various dimensions that constitute their identity. The current push for disability inclusion highlights that, while inclusive education and calls for “Education for All” refer to all learners, those with disabilities have continued to be structurally marginalised even within movements towards inclusion (Miles and Singal 2010). Because of this, our discussion of inclusive refugee education seeks to achieve inclusive education for all learners, highlighting the particular challenges faced by students with disabilities who have historically been deprioritised in theory and practice.

Inclusive quality education is axiomatic to the building of just societies. As such, movements towards inclusive education must be based on principles of social justice rather than charitable approaches (Singal et al. 2017). For the purposes of this article, we will engage with how the logic of refugee education exacerbates the exclusion of learners with disabilities on three levels: entry, engagement, and empowerment (Singal 2014). “Entry” concerns access to schools; “engagement” refers to matters such as the availability of appropriate resources and teacher skills; and “empowerment” refers to the development of agency and the fostering of a sense of belonging (ibid.). To achieve inclusion, all three levels must be addressed.

This article offers a conceptual reflection, emerging from or own research in the field as well as our engagement with the literature. We discuss four critical tenets of refugee education. First, we historicise the emergence of the modern system of refugee education, highlighting how it has been driven by the security interests of Western states, and the colonial logic intrinsic to the broader humanitarian project. Refugees with disabilities, who fall outside constructs of the “threatening migrant”, trouble this notion of securitisation and the “ideal student” of refugee education programmes. Second, refugee education is guided by neoliberal assumptions, with two important implications: funding structures are guided by ideals of cost–benefit analysis, and schools are geared towards notions of future employability. This outlook portrays refugees with disabilities as more difficult/costly and less valuable to include.

Despite overarching structures that facilitate marginalisation, there has been a positive trend to include refugees with disabilities in education. However, the logics used to conceptualise these efforts lead to further marginalisation. This brings us to our third point, which is that refugee education appropriates a charitable logic, relying on the language of vulnerability to classify refugees with disabilities, undermining movements towards inclusive education. Fourth, we highlight the conflicting temporal pressures at play between “education”, “humanitarianism” and “disability inclusion”. The logic of short-termism and the presumed temporary nature of the refugee situation lead to a disregard for an “unknowable future” (Dryden-Peterson 2017) and an emphasis on the present. This has two important implications: (1) organisations find it easier to justify the “temporary” exclusion of refugees with disabilities, and (2) pedagogically, the focus of education is placed on filling time, rather than engaging and empowering students.

While these four logics are neither comprehensive nor exhaustive, we highlight them in our article given their persistent emergence throughout our research as organising ideas that shape how refugee education is conceptualised and implemented.

## The logic of securitisation

While history has seen a variety of conceptualisations of the purposes of refugee education (see for example Chatty 2017), our analysis focuses on the predominant Western framework which has been largely responsible for defining education responses in the Global South, guiding the marketisation and politicisation of aid, as well as refugees’ experiences with education. Humanitarianism functions as an extension of the colonial order (Chimni 2009) and an instrument to serve its continuation (Aloudat and Khan 2022), reproducing political and economic inequalities and unequal power relationships between the Global South and the Global North (Saleem 2023). Refugee education, as a subset of the broader humanitarian project, is inscribed in this neocolonial effort.

The past few decades have seen a growing interest in the field of refugee education in the policies of Western governments and Western-dominant international actors. This has sprung from a growing awareness of the increasing number of out-of-school children in crisis-affected contexts, and from a belief that lack of education may exacerbate instability in fragile countries, with repercussions on global security (Burde et al. 2017). In the aftermath of 9/11, Western governments saw countries affected by conflict as a threat to their internal and global stability (Barnett 2009). Consequently, at the beginning of the 21st century, dominant refugee education interventions, including the funding and sustenance of research, came to be defined in terms of securitisation, aimed at preserving Western interests and agendas (Burde et al. 2017, 2019; Novelli 2010; Winthrop and Matsui 2013). The process of securitisation tends to construe an issue, a group or a phenomenon as a national or global security concern, prompting the use of extraordinary measures of control. This is carried out through discourses and policies driven by governments, media and prominent institutions (Buzan et al. 1998).

Securitisation, as an underlying theorising feature of refugee education, contributes to processes of “otherisation”, fuelling the imaginary notion of the “threatening migrant” (Fassin 2012; Massari 2021). This approach has tangible repercussions on the quality, nature and practices of educational provision in areas of conflict. The heavy securitisation of the refugee education sector implemented post-9/11 does not naturally fit with a philosophy of inclusive education. For instance, people with disabilities who are not framed as a “security threat” disrupt the notion of the threatening migrant, as they do not comfortably fit within the construction of the “threatening other” and the attendant rationale of education-for-securitisation. Likewise, the theorisation of education as either a driver of or a remedy against conflict (Bush and Saltarelli 2000) conceptually links education with violence, with the implicit assumption of able-bodied, conflict-affected persons who are capable of such violence. This presumption of the able-bodied, threatening refugee goes hand-in-hand with the presumed vulnerability of refugees with disabilities, discussed below, which discursively construes them as weak and docile, and probably incapable of posing a security threat. As a result, people with disabilities have been largely excluded from theory and practice, falling outside the imaginations of students who benefit from education under the auspices of securitisation concerns. Inclusive education is concerned with *entry* into education as a first step; the logic of securitisation creates challenges to entry for refugees with disabilities, excluding them from the very beginning.

It may be true that individual humanitarian actors do not consciously adopt a logic of securitisation when designing and implementing refugee education programmes. Yet they continue to be implicated subjects (Rothberg 2019; see also Novelli and Kutan 2023) within a system that has been designed with security interests in mind, into which refugees with disabilities do not comfortably fit. In order to move away from this, it is critical for the field of refugee education to critically examine its relationship with colonialism and harmful logics of securitisation in both theory and practice.

## The logic of neoliberalism

Neoliberalism is an economic and political philosophy which emphasises the value of free market competition, the liberalisation of trade, deregulation of labour and more generally the commodification of various, if not all, aspects of life (Gilens 2014). In humanitarian contexts, neoliberalism explains the influence of free-market principles on the delivery and organisation of humanitarian aid. The logic of neoliberalism within refugee education shapes its assumptions and implementation, and facilitates the exclusion of refugees with disabilities from education and humanitarian practice more broadly (Grech 2015; Trani et al. 2012; Chaudhry 2015). For instance, the donor–implementor relationship becomes a site for tension, as

the push for quick outcomes demanded by donors makes disabled people an unattractive proposal, especially since these require a long-term commitment and resources, and the results may not be immediately evident (Grech 2011, p. 96).

Within the frame of neoliberal humanitarianism, disability is commodified, medicalised, and constituted as an “economic problem” (Berghs 2014). This goes against the fundamentals of inclusive education, prioritising the interests of the “humanitarian market” rather than the needs of the people it purports to support.

This neoliberal logic is most evident in the “cost-benefit analysis that determines who should be educated” (Trani et al. 2012, p. 20), leading to the marginalisation of people with disabilities compared to those without, and to a hierarchisation of disability based on the financial costs of integration. Adopting this paradigm, actors seek to invest the minimum resources to reach the highest quantifiable outcomes, often because they are competing for continued funding from donors who emphasise quantitative metrics. This utilitarian logic interacts with ableist assumptions and prioritises the education of those deemed least costly to include, clearly conflicting with the principles of social justice that underlie inclusive education.

When the international organisations (IOs) and NGOs in our research who design disability-inclusive refugee education programmes in Lebanon discuss their rationale, they emphasise the costs of including people with different kinds of disabilities. One IO representative explained that those with mild intellectual disabilities such as attention-deficit hyperactivity disorder (ADHD) and autism are the easiest to include because they do not require substantial material investments. She contrasted them with wheelchair users, who would require infrastructural changes to their schools, and people with sensory impairments, who would require specialised learning material. Importantly, she discussed the resistance of teachers and parents of other students to include learners with moderate and severe disabilities, which would incur the additional costs of teacher training and attitudinal change. However, this cost–benefit analysis is not always calculated in the same way. A representative of a Lebanese OPD explained that the installation of a ramp for wheelchair users is the easiest measure to take to include people with disabilities, whereas integrating people with intellectual disabilities is more difficult. When refugee education actors discuss their reasoning for including or excluding students with disabilities, they always forefront the material and human costs involved as the main factor determining whom to include. This leads to a hierarchisation of disability, whereby those deemed least costly to include are prioritised. The homogenisation of “disability inclusion” reporting means that those not included are further marginalised in reporting and planning.

In addition to the costs associated with inclusion, the likelihood of future employability also constitutes a determining factor in prioritising who to include. This interacts with ableist assumptions that refugees with disabilities are unlikely to pursue future employment. With the prevalence of this logic, a learner’s capacity to fit within neoliberal and ableist normative standards of productivity and efficiency represents a fundamental requirement for their inclusion. Interestingly, while education providers do not generally discuss their education programmes in relation to employment, when they discuss their reasoning for excluding and deprioritising refugees with disabilities, low employment prospects are consistently raised as an issue.

The reduction of disability-inclusive education to economised metrics reinforces the medicalisation of disability, locating it in individual deficit, and creates a sterile understanding of “inclusion” reducible to the installation of ramps and other material resources. This transforms inclusion into a “tick-box exercis[e]” (Lough et al. 2022, p. 16) and takes the focus away from a serious shift towards sustainable inclusive practices that target entry, engagement and empowerment, encompassing both the attitudinal and material changes required for inclusion. In response to donor speed, inclusive education projects in displacement contexts prioritise short-term interventions at the expense of longer-term projects. The desire to report on higher numbers of children “included” leads to the prioritisation of the mildest instances of disability and the most cost-effective choices for inclusion. As a consequence, projects are labelled as being disability-inclusive, while many learners with disabilities continue to be explicitly excluded because they are too costly to include or too far removed from neoliberal tropes of productivity and employment.

In a similar vein, competition for donor funds leads to the hyper-visualisation of reporting on disability inclusion. Local and international organisations exaggerate their reporting on disability inclusion through visuals, which may not comprise the most effective approach to inclusion. A clear example was the installation of ramps by a large IO in its schools as part of an inclusive education project. When probed on why this measure was taken, it became clear that the organisation did not know whether any students actually required a ramp. Such visual interventions respond to the competition for donor funding and may be prioritised at the expense of practices that are less visually appealing, yet more effective in supporting inclusion. Refugee education providers may uncritically over-report on success cases of “easy inclusion”, leveraging “(dis)ableist imagery” to solicit pity and funding (Berghs 2014, p. 35), whilst remaining structurally exclusionary.

The framing of inclusive refugee education through a neoliberal prism is reproduced at the macro level by donors, IOs and NGOs, at the meso level by school personnel, and at the micro level by families of refugees with disabilities. When caregivers have insufficient resources to send more than one child to school, they often prioritise those without disabilities, citing their likelihood of future employability as the reason. When discussing their reasoning for sending their children to school or keeping them at home, caregivers very often refer to the difficulties people with disabilities face in gaining employment, concluding that there is no point in sending them to school.

However, while the neoliberal logics of cost and future employability are highlighted by most actors in the refugee education ecosystem, they are usually not the highest priority of refugees with disabilities themselves. In our research, refugees with disabilities took proactive steps in explaining to schools and their families how they could participate in learning without financial investment, arguing that the cost should not be a barrier to their participation. For example, blind students often record the lesson on their phones in lieu of making notes so that they can study from the recording at home, ask teachers to speak aloud while writing on the board so they can capture the lesson content, or ask their peers to read to them from textbooks. Moreover, many refugees with disabilities in our research considered themselves to have bright employment prospects and had clear career paths they intended to pursue. They also refused to conceptualise education purely in terms of employment, outlining other benefits of going to school, such as socioemotional benefits, developing a sense of belonging, and forging friendships.

## The logic of vulnerability

Vulnerability within humanitarian contexts is typically defined as the risk of exposure to harm and the inability of populations or individuals to deal with the consequences of that harm (e.g. UNHCR 2022). Refugees with disabilities are often classified as vulnerable by virtue of having disabilities. While the refugee education logic seeks to identify students deemed as vulnerable in order to protect them, this logic has been criticised for its use as a mechanism of humanitarian governance and control which disempowers communities deemed “vulnerable” and solidifies hierarchical and unequal power dynamics (Turner 2021; Sözer 2021). In line with this critique, we argue that the uncritical use of vulnerability logics to define and categorise refugees with disabilities serves to disempower students and reinforce harmful stereotypes even when they are included in education spaces.

In Arabic, “vulnerable” is translated as “*mustadaaf*”,^2^ which is the same word for “weak” and “disempowered” and carries negative connotations. This classification places students with disabilities in an inferior position to their peers (El-Serafy, forthcoming-b) so that even when access to schools is achieved, the goal of empowerment is jeopardised. Our participants rejected the label of vulnerability and asserted instead their need for dignity, diversity and inclusion, arguing that these conflict with the disempowering discourse of vulnerability. The field of disability studies has largely moved away from such discourses, critiquing charitable and vulnerability-focused approaches to disability which deny equitable individual rights and challenge the construction of actors as protectors when they are complicit in exerting violence (Gill and Schlund-Vials 2014; Kim 2014). The use of vulnerability assessments in humanitarian aid more broadly has similarly been critiqued for depersonalising individuals and denying them hope for the future (Brun 2016). In international humanitarian law, the use of vulnerability discourses conflicts with social justice-based ideals of inclusion, and highlights the reliance of humanitarian law on medicalised approaches to disability (Lord 2014). In the logic of refugee education, disability continues to be uncritically deemed a determining factor for vulnerability.

The vulnerability rhetoric hampers inclusion when it is attached to specific types of bodies, making their designation as vulnerable reductive and inescapable regardless of an individual’s societal, environmental and affective circumstances. The association of vulnerability with disability has long been critiqued in disability studies literature (El-Bushra and Gardner 2016). The process of ascribing vulnerability places the disability on the individual rather than in society, once again evoking outdated, medicalised understandings of disability and neglecting the national and transnational processes which create the inequalities experienced by those with disabilities (Berghs 2014). This leads to inclusive education efforts which focus on the individual’s impairment, such as installing ramps, rather than working towards attitudinal and societal adjustments to make school environments more inclusive, such as training teachers, parents and students on the importance of diversity and inclusion.

Refugees with disabilities exhibit a broad spectrum of preferences, skills, life experiences and hopes for the future, which are not captured within the essentialist and reductive vulnerability rhetoric leveraged by education providers who are often ill-equipped to meet refugees with disabilities where they are in their lives. For instance, Maysa,^3^ the mother of a young girl with a disability, explained how there were no educational opportunities for children with moderate to severe disabilities like her daughter. Despite her efforts to enrol her child in school, she reported that “there is no integration for children with disabilities”. She felt sure that if her daughter were given a chance to be educated, “she will be able to do great things. But unfortunately, no one is accepting her, no one is ready to see her potential and to integrate her” (Costantini 2025, p. 192). Seeing Maysa’s daughter, and others in her position, as *only* vulnerable prevents education actors from seeing their potential.

Importantly, while international actors in the field of refugee education continue to construe those with disabilities as vulnerable, refugees with disabilities themselves reject this label. Even outside the space of disability, refugees typically reject the label of vulnerability imposed on them by humanitarian actors (Turner 2021; Carpi and Mourad, forthcoming; Carpi 2023), a trend which is typical of the categories used in humanitarian work (for example Rutazibwa 2019; Agier 2011; Zetter 1991). This extends to refugees with disabilities who, living in a context that denies their agency, reject this categorisation as an affront to their capacities.

Besides the issue of imposing hierarchical labels that populations themselves do not identify with, the discursive construction of refugees with disabilities as vulnerable works against the goal of empowerment within and through inclusive education. This tension draws on the inherent conflict between construing disability as vulnerability or as diversity (Erevelles and Nguyen 2016). As commentators on humanitarianism note, “[h]umanitarian categories tend to fix people in particular places and social positions” (Brun 2016, p. 394). The discursive determination of subjects as vulnerable consolidates and reproduces their vulnerability, rather than facilitating diversity and inclusion (Kim 2014) and cements harmful power dynamics within humanitarian work which perpetuate asymmetrical power relations (Cole 2016). The discourse of vulnerability denies the agency of individuals, portraying them instead as passive and in need of protection (Berghs 2014). As a consequence, it falls short of the “empowerment” aims of inclusive education.

In Lebanon, people with disabilities face widespread stigma (HRW 2018; Combaz 2018; Marouche et al. 2021). A report published in 2020 highlighted that teachers “did not believe all students with disabilities could be successfully included” in education (GEMRT 2020, p. 136). Participants in our research emphasised that they often had to work against their own internalised limiting beliefs, in addition to those emanating from their families, teachers, school leaders, peers and aid workers. A blind participant in our research was 16 when he first learned that blind people could go to school. He enrolled himself in an accelerated learning programme and was consistently scoring top of his class. Despite this, his family, teachers and peers often advised him to drop out, since they thought learning would be too difficult for him due to his blindness. He relayed how this led him to feel angry and to doubt himself. He felt his achievements were not taken seriously because people only saw his disability. While they were trying hard to assert their rights to education and their skills, our participants emphasised that labelling them as vulnerable is more limiting than helpful. The discourse of vulnerability within refugee education may set out to protect the communities it identifies as vulnerable, but it risks working against this goal by maintaining and reproducing positions of disempowerment within schools and society.

## The logic of time

Surviving in the present and planning for a future represent a clashing of temporalities in a context where the humanitarian system and people living in and with crisis (conflict, disaster and displacement) envisage futures differently (Brun 2016, p. 394).

Cathrine Brun (2016) discusses the temporal dimensions of humanitarian work, highlighting the conflicting temporal pressures at play between humanitarian actors seeking to “save lives” in the present and people in contexts of displacement seeking to plan for their futures. She argues that “humanitarian action primarily aims for temporary solutions that tend to make people stuck in a humanitarian system for years” and that “there is little room for thinking about futures in the current humanitarian system” (Brun 2016, p. 394). The “future” is understood here as a “temporal dimension of experience” that adds meaning to the past and present (ibid., p. 401) and may encompass aspirations, goals and hopes related to an individual’s professional, academic, social and personal life.

Critiquing the temporal contradictions in the framework of emergency education in Lebanon, which is host to a protracted refugee context, Cathrine Brun and Maha Shuayb (2020) argue that the emergency framework of “Education in Emergencies” (EiE) emphasises the present, while education is a developmental, future-oriented activity. In our research in Lebanon, we found these temporal politics playing out in two distinct ways to exacerbate the marginalisation of refugees with disabilities. First, humanitarian actors often used the reasoning of emergency and temporariness to justify the exclusion of those deemed too difficult to include. Second, with the focus of education on saving lives in the present, initiatives targeting refugees with disabilities often aimed to occupy their time in the present, rather than their future development. While humanitarian actors were present-oriented, refugees with disabilities in our research often resisted this, and sought out ways to plan for their futures.

When interviewing humanitarian actors and NGO representatives about their inclusive education policies, we found that they often leveraged the rhetoric of emergency and temporariness to justify the exclusion of refugees with disabilities. They explained that including people with disabilities would require a long-term, multi-pronged process of developing accessible resources, conducting attitudinal change workshops, and making adjustments to school buildings. However, refugees with disabilities who had previous experience of joining mainstream refugee education programmes said that these adjustments were not necessary and that they preferred to join school without them rather than remaining out of school. Similarly, this rhetoric was leveraged by humanitarian actors to explain their hierarchisation of people with different kinds of disabilities, prioritising those with mild disabilities who would be quicker to include. This discourse interacts with the precarity of the future, with some actors explaining that they were unwilling to implement long-term processes of inclusive education since they did not know whether refugees would eventually leave the country. In this way, the “logic of emergency prioritises the system over the individual” (Gatter 2023, p. 4).

The pervasive logic of temporariness is similarly leveraged by refugee education actors to portray the marginalisation of learners with disabilities as more acceptable. Since they are responding to an emergency, construed as impermanent, the marginalisation of learners with disabilities is, likewise, impermanent. This makes the exclusion seem more acceptable, and allows refugee education actors to profess their commitment to inclusive education *eventually*, even as they do not implement it now. While this attitude was used to explain the choice not to include refugees with disabilities in education programmes by mainstream humanitarian actors, some initiatives in Lebanon specifically targeted refugees with disabilities. In these cases, we saw conflicting temporal logics between education providers who focused on the present and students who wanted to plan for their futures. Education providers tended to emphasise the precarity of the future, as well as harbouring ableist assumptions on what refugees with disabilities would be able to achieve in future. Accordingly, their focus was on occupying students’ time in the *present*, rather than being developmental and future-oriented. There were multiple instances when interlocutors in our research, from OPDs, IOs and NGOs, heralded the success of instances of “inclusive education” when students were present in classrooms but not allowed to engage with any learning material, examination processes or in-class teaching at all.

For example, one of the students we saw was a deafblind woman in her early 20s who attended a school run by an OPD that targeted blind refugees. Because she was deaf as well as blind, the teachers did not know how to interact with her, teach her, or include her in learning. Instead, she would sit in the corridor alone and find something to occupy herself with, such as weaving baskets or making jewellery. When the teachers spoke about her, they heralded her case as one of successful inclusion, despite her not being engaged in learning.

When discussing with school leaders the reasoning behind their choice of curriculum, teachers often explained that they chose options that were fun, that would fill the students’ time, and that would get them out of their homes. Their explanations were mired with ableist assumptions, including the belief that people with disabilities could not study or work anyway, so there was no reason to prepare for their futures. Critical issues such as certification and progression were sometimes disregarded entirely, as they were seen as only necessary for the “future”. With the exception of a few programmes which provided year-long learning for refugees with disabilities, most educational initiatives aimed at them were fragmented and ad-hoc one-day activities, such as workshops on soap-making or photography. These come at the expense of future-oriented, developmental activities which recognise and cater to students’ needs in terms of skills, knowledge and socioemotional development.

## Conclusion

The field of refugee education is poorly theorised, and efforts to include refugees with disabilities in theorising have hitherto been based on conflicting logics. Refugees with disabilities are caught between a disempowering politics of pity and a troubling politics of securitisation. These two logics are antagonistic to each other and result in an uncritical, homogenous construal of refugees with disabilities.

In our research, we have encountered many well-meaning efforts from international and local Lebanese NGOs who seek to include refugees with disabilities, but who find themselves working within a humanitarian system whose logic does not support inclusive education encompassing all three levels of entry, engagement and empowerment. As efforts to theorise refugee education gain traction, it is important to consider ways to engage in cross-disciplinary dialogue so that the field of refugee education can learn from the rich traditions of other fields that have advocated for inclusion, so as to develop a more socially just and inclusive model of refugee education for all learners.

Beyond securitisation logics, cost-effective and quota-driven tokenism, ableist notions of vulnerability and erasure of future hope, there lies a space where refugee education can be truly inclusive of learners with disabilities. Refugee education is too important to abandon. In order to grow as a field, it needs to engage in a critical reckoning with the colonial, neoliberal and ableist assumptions that currently inform theory and practice. When we start to listen to the voices of refugees with disabilities, we will find that they offer many solutions to the problems of inclusion that the field of refugee education continues to grapple with.

## Footnotes

1 Whilst we use the term “refugees”, we recognise that some people are forced into displacement through irregular routes and may thus fall outside legal definitions of what constitutes a refugee.

2 Author’s translation.

3 A pseudonym.
